# Early sport specialization and youth athlete health: a structured narrative review of injury, training load, movement and neuromuscular adaptation, and psychological well-being

**DOI:** 10.3389/fspor.2026.1945833

**Published:** 2026-10-22

**Authors:** Ethan M. Choi, Cynthia van Golen

**Affiliations:** 1 Santa Margarita Catholic High School, Rancho Santa Margarita, CA, United States; 2 Department of Biological Sciences, Delaware State University, Dover, DE, United States

**Keywords:** athlete development, early sport specialization, euromuscular adaptation, overuse injury, psychological well-being, training load, youth athlete, youth sport

## Abstract

**Background:**

Early sport specialization (ESS) has become increasingly common in competitive youth sport, although its benefits for long-term athletic development and consequences for athlete health remain uncertain. Specialization frequently co-occurs with greater training volume, year-round participation, repetitive loading, and reduced recovery, complicating its interpretation as an independent risk factor.

**Objective:**

This structured narrative review synthesizes evidence concerning ESS and youth athlete health across injury, training exposure, movement and neuromuscular adaptation, physical performance, and psychological well-being.

**Methods:**

A structured PubMed/MEDLINE search identified relevant literature published primarily between January 2015 and May 2025. Reference lists and previous reviews provided additional contextual and foundational evidence. Studies were organized thematically, with particular attention to factors that might confound or modify associations attributed to specialization.

**Results:**

Specialization was associated with increased injury risk in several youth athlete populations, particularly for overuse injuries, although associations varied across sports and athlete subgroups. Adjustment for training volume attenuated these associations in some studies, suggesting that workload may partly explain observed relationships. Movement and neuromuscular findings were task-specific and did not establish a uniform pattern of impaired movement competency. Psychological evidence was comparatively limited and heterogeneous, with outcomes influenced by training demands, performance pressure, recovery, and the broader sport environment.

**Conclusions:**

ESS should not be considered an isolated exposure or universal determinant of adverse outcomes. Athlete-development recommendations should account for cumulative workload, recovery, maturation, sport-specific demands, and psychological well-being. Prospective studies separating specialization from correlated training exposures are needed.

## 1 Introduction

Youth sport participation provides substantial opportunities for physical development, skill acquisition, social engagement, and long-term involvement in physical activity. However, as competitive opportunities have expanded, the developmental pathways young athletes follow have also changed. One increasingly prominent pathway is early sport specialization (ESS), in which young athletes concentrate their participation and training within a primary sport rather than maintaining involvement across multiple sports. Specialization may emerge in response to athlete preference, competitive opportunities, coaching structures, parental investment, perceived scholarship or recruitment advantages, or the belief that accumulating sport-specific practice at younger ages is necessary for later athletic success (1–4).

Despite widespread use of the term, sport specialization has not been defined consistently. Contemporary consensus defines sport specialization as intentional and focused participation in a single sport for a majority of the year that restricts opportunities for engagement in other sports and activities (5). Most empirical studies similarly conceptualize specialization as intensive participation in a single primary sport, often accompanied by year-round training and the exclusion or discontinuation of other sports (6–8). The concept of early specialization adds a developmental dimension, implying that this concentrated participation begins during childhood or early adolescence. However, the age or developmental stage considered “early” varies among studies, and some investigations classify specialization according to current participation patterns rather than age at specialization. Other studies operationalize the exposure using months per year in a primary sport, single-sport status, cessation of other sports, or combinations of these characteristics. These differences are not merely semantic; they affect which athletes are classified as specialized and complicate comparisons across studies (9, 10).

The rationale for sport specialization is often linked to the intuitive expectation that greater sport-specific practice will accelerate skill development. Deliberate-practice theory has strongly influenced discussions of expertise, emphasizing the importance of sustained, purposeful practice for attaining high levels of performance (11). However, deliberate practice does not necessarily require early exclusive participation in one sport, nor does it establish a universal amount of childhood training required to achieve elite performance. Athlete-development frameworks similarly recognize that pathways to expertise may differ among sports and individuals. The Developmental Model of Sport Participation emphasizes early sampling and diversified participation as one pathway toward later specialization, while consensus recommendations generally encourage broad participation during childhood and more gradual progression toward intensive sport-specific training (8, 12). Accordingly, the relevant developmental question is not simply whether sport-specific practice is necessary for expertise, but when increasing specialization becomes advantageous, neutral, or potentially detrimental for a particular athlete and sport.

Concern regarding ESS has centered primarily on athlete health and on whether early concentrated participation meaningfully improves later athletic performance. Professional organizations and previous reviews have associated highly specialized or intensive youth-sport participation with overuse injury, burnout, and other adverse outcomes and have consequently recommended caution regarding year-round single-sport participation (7, 8, 13, 14). Injury has received the greatest empirical attention. Bell et al. (6), for example, reported in a systematic review and meta-analysis that highly specialized youth athletes had greater risk of overuse injury than athletes with low specialization. Carder et al. (15) similarly found, across six studies involving 5,736 youth athletes, that sport sampling was associated with lower injury risk than sport specialization. In contrast, evidence that earlier specialization improves subsequent athletic performance is substantially less convincing. Kliethermes et al. (16), in a systematic review of career- and task-specific performance outcomes, concluded that youth sport specialization was not required for elite athletic success and did not appear to improve task-related performance during childhood or adolescence. Together, these previous syntheses suggest that the health implications of specialization should be considered alongside an uncertain performance benefit rather than assuming that greater early sport-specific investment necessarily produces superior long-term athletic outcomes. Subsequent studies have continued to identify associations between specialization and injury in selected youth populations, including associations involving overuse injury and sport-specific injury patterns (17–22).

However, interpreting these associations as evidence that specialization itself causes injury presents an important methodological problem. Sport specialization rarely occurs in isolation. Highly specialized athletes may train for more hours per week, participate in their primary sport for more months each year, compete on multiple teams, experience fewer seasonal breaks, and accumulate greater exposure to repetitive sport-specific movements. Specialization may therefore correlate with several exposures that can influence injury independently. Studies support this distinction: adjusting for activity or training volume attenuates the association between specialization and injury, while other studies identify independent associations for both specialization and workload-related variables (17, 20, 22, 23).

Training exposure itself is also a heterogeneous construct. Studies have quantified exposure using weekly vigorous physical activity, organized sport hours, primary-sport training hours, months of participation per year, annual sport hours, or athlete-exposures. These measures represent related but distinct characteristics of an athlete's training environment and should not automatically be treated as equivalent measures of physiological workload. Similarly, the commonly used criterion of participating in a primary sport for more than eight months per year may reflect annual exposure, reduced seasonal recovery, or specialization depending on the study definition. Separating these constructs is therefore essential when evaluating whether specialization independently contributes to adverse outcomes.

This conceptual overlap suggests that specialization may be useful not only as an individual exposure variable but also as a potential exposure phenotype. Highly specialized athletes may share some combination of greater sport-specific volume, year-round participation, repetitive movement exposure, fewer opportunities for seasonal recovery, higher competitive demands, and greater investment in a primary athletic identity. Importantly, the composition of this phenotype is unlikely to be identical across athletes or sports. A specialized baseball pitcher, gymnast, swimmer, soccer player, basketball player, or fencer may experience substantially different mechanical and developmental exposures despite receiving the same categorical specialization classification. This heterogeneity could help explain why associations between specialization and adverse outcomes differ across sports and athlete populations, and why statistical adjustment for training exposure attenuates associations in some studies but not others.

Movement and neuromuscular development represent another area that requires cautious interpretation. Single-sport and highly specialized athletes have demonstrated differences in balance, landing technique, coordination, joint coupling, and other movement characteristics (24–29). Yet a measurable difference between specialized and nonspecialized athletes does not necessarily represent dysfunction. Movement variability, asymmetry, or sport-specific coordination may reflect adaptive specialization, developmental variation, task-specific strategies, or potentially maladaptive patterns depending on the context. Establishing clinical significance therefore requires more than demonstrating between-group biomechanical differences; ideally, such characteristics should be linked prospectively to subsequent injury or performance.

Similar caution is necessary when considering psychological and psychosocial outcomes. Burnout, stress, athletic identity, parental influence, reduced time away from sport, and mental-health concerns have all been discussed in relation to intensive youth-sport environments (30–32). However, the direct empirical literature isolating specialization from training demands, competitive pressure, recovery opportunity, parental involvement, and the broader sport environment remains considerably smaller than the injury literature. Consequently, psychological outcomes observed among intensively involved athletes should not automatically be attributed to ESS unless specialization itself has been evaluated.

Sport-specific mechanical demands add another layer of complexity. Repetitive throwing in baseball, high-volume jumping in volleyball, repetitive technical training in gymnastics, and dominant-limb or laterality-dependent movements in other sports represent substantially different exposures. The same specialization classification may therefore have different implications depending on the sport, position, event, sex, maturation status, and developmental context. This issue is especially relevant for understudied sports with distinctive repetitive or lateralized movement demands. For example, fencing has well-characterized physiological, biomechanical, and injury demands (33–35), but the current literature does not establish that specialization itself produces these injury or biomechanical patterns. Such sports consequently represent important opportunities for future specialization research rather than evidence of an established ESS effect.

Taken together, these limitations suggest that the traditional question of whether ESS is simply “harmful” or “safe” may be too narrow. Specialization occurs within a multidimensional developmental environment encompassing cumulative training exposure, recovery, sport-specific loading, biological maturation, sex, previous injury, competition level, athlete goals, and psychosocial context. Understanding youth-athlete health therefore requires determining not only whether an athlete specializes, but also what pattern of exposure and developmental conditions that specialization represents.

Accordingly, the purpose of this structured narrative review was to synthesize contemporary evidence concerning early sport specialization and youth athlete health across four interconnected domains: (1) injury and overuse injury; (2) sport specialization, training volume, and workload; (3) movement and neuromuscular adaptation and physical performance; and (4) psychological and psychosocial well-being. Particular attention was given to distinguishing specialization from correlated training exposures, identifying sport-, sex-, and developmental-stage differences, and differentiating direct ESS evidence from contextual biomechanical, psychological, and sport-specific literature. By examining specialization within this broader developmental framework, this review aims to identify both what can presently be concluded about ESS and where apparent specialization-related effects may instead reflect or interact with the wider environment of intensive youth sport.

## 2 Methods

### 2.1 Review design

This study was conducted as a structured narrative review examining early sport specialization (ESS) and youth athlete health. We used a structured literature search and thematic synthesis to identify and organize contemporary evidence on injury and overuse, training exposure, movement and neuromuscular adaptations, athletic development, and psychological and psychosocial outcomes.

The review was designed to provide an integrative synthesis across these interconnected domains rather than an exhaustive systematic identification of all potentially eligible studies. Accordingly, the review was not prospectively registered and does not claim adherence to PRISMA systematic-review methodology. The structured approach was retained to improve transparency regarding how the literature informing the narrative synthesis was identified and interpreted.

### 2.2 Literature search

We conducted a structured literature search in PubMed/MEDLINE to identify relevant peer-reviewed literature on early sport specialization and youth athlete health. The primary evidence window was January 1, 2015 through May 31, 2025. The final search strategy was reconfirmed in PubMed/MEDLINE on September 2, 2026, using the same publication-date window and returned 213 records. We selected PubMed/MEDLINE because it covers sports medicine, pediatrics, orthopedics, exercise science, and related biomedical disciplines.

Search terminology incorporated combinations of terms addressing sport specialization, youth athletes, injury, training exposure, movement and neuromuscular outcomes, performance, and psychological outcomes. The principal search framework was: (“sport specialization” OR “sports specialization” OR “early specialization” OR “single-sport participation” OR “single sport”) AND (youth OR adolescent* OR child* OR pediatric OR athlete*) AND (injury OR overuse OR training OR workload OR burnout OR psychological OR psychosocial OR “mental health” OR biomechanics OR development OR performance).

Results were limited to English-language publications. Because the search aimed to support a structured narrative synthesis rather than exhaustively identify all eligible studies, records were screened for relevance to the principal domains of injury and training exposure; movement, neuromuscular, and performance outcomes; psychological and psychosocial outcomes; and sport-specific or developmental context.

We also examined reference lists of relevant primary studies and previous reviews to identify publications providing important contextual, methodological, or mechanistic information. We also used relevant systematic reviews to contextualize the primary evidence base and identify potentially relevant original studies, and we distinguished review findings from the individual empirical studies summarized in the evidence tables. We retained earlier foundational publications where necessary to describe the development of sport-specialization definitions, deliberate-practice concepts, athlete-development frameworks, injury mechanisms, or professional recommendations.

### 2.3 Selection and use of evidence

We prioritized empirical studies directly examining sport specialization or closely related patterns of single-sport participation in relation to injury, training exposure, movement and neuromuscular outcomes, athletic performance, or psychological and psychosocial outcomes.

We emphasized studies involving children and adolescents. The review considered studies involving collegiate or adult athletes selectively when they retrospectively assessed sport-specialization histories during childhood or adolescence. We identified such studies as retrospective evidence rather than as studies of current youth populations.

The review also incorporated selected contextual literature. We used injury-surveillance, biomechanical, and sport-specific studies that did not directly evaluate specialization only when they informed interpretation of mechanisms, sport-specific exposures, or research gaps. We distinguished these publications from studies that directly evaluated relationships between specialization and athlete outcomes.

We used previous systematic reviews, narrative reviews, consensus statements, and professional position statements to establish the existing evidence base, describe conceptual frameworks, and contextualize recommendations. We did not treat them as independent primary evidence when evaluating specialization-outcome associations.

This distinction was particularly important for sport-specific literature. Studies describing repetitive loading, injury epidemiology, or biomechanical demands within a sport were not interpreted as demonstrating an effect of ESS unless specialization itself was measured.

### 2.4 Evidence organization and extraction

We organized relevant study information using a structured evidence-extraction table. Extracted variables included, where available:

- Study design and population;
- Participant age and sex;
- Sport or sports examined;
- Definition or operational measure of specialization;
- Training volume or annual exposure;
- Comparison group;
- Injury definition and denominator;
- Movement, neuromuscular, performance, or psychological outcomes;
- Quantitative effect estimates and 95% confidence intervals;
- Covariates included in statistical models; and
- Principal findings and interpretive limitations.

We paid particular attention to variables that could confound or modify associations attributed to specialization, including weekly training volume, months per year of participation, previous injury, sex, biological maturation, competition level, and sport-specific demands.

We recorded definitions of specialization where available because substantial heterogeneity exists across the literature. This review used the contemporary consensus definition proposed by Bell et al. (5) as its conceptual framework, while recognizing that many earlier studies operationalized specialization using different combinations of single-sport participation, year-round participation, cessation of other sports, or training duration.

### 2.5 Thematic synthesis

Evidence was synthesized narratively across four interconnected domains:

1. Injury and overuse injury;
2. Sport specialization, training volume, and workload;
3. Movement and neuromuscular adaptation and physical performance; and
4. Psychological and psychosocial outcomes.

We considered sex-, sport-, and developmental-stage differences where reported but did not treat them as formal subgroup analyses. We retained sport-specific findings when they materially altered interpretation, rather than collapsing them into a single generalized specialization effect.

Where available, we reported quantitative effect estimates and 95% confidence intervals to convey the magnitude and precision of associations. We paid particular attention to estimates adjusted for training or activity volume because of the substantial conceptual and empirical overlap between specialization and cumulative training exposure.

We reported biomechanical findings according to the variables actually measured. We did not automatically classify differences in movement variability, coordination, balance, joint coupling, or other biomechanical outcomes as beneficial or detrimental unless the original study directly linked the observed characteristic to injury or performance.

Psychological evidence was similarly differentiated according to source type. Empirical studies directly evaluating specialization-related psychological or psychosocial outcomes were distinguished from broader youth-sport studies, reviews, and position statements used to provide contextual interpretation.

### 2.6 Interpretation of evidence

This structured narrative review did not include a formal meta-analysis, systematic risk-of-bias assessment, or GRADE certainty assessment. Therefore, evidence was not assigned categorical ratings such as “strong,” “moderate,” or “limited.”

Interpretation instead considered study design, magnitude and precision of reported associations, statistical adjustment, consistency across studies, and important sources of heterogeneity and confounding. We applied particular caution when interpreting observational associations as causal relationships.

We did not assume specialization represented the operative exposure when training volume, annual participation, recovery, or sport-specific loading could plausibly explain the observed associations. When studies adjusted for these factors, we prioritized adjusted estimates in the narrative interpretation.

## 3 Evidence synthesis

### 3.1 Sport specialization, training exposure, and injury

The relationship between sport specialization and injury represents the most extensively studied health outcome in the literature reviewed here, but the evidence does not support a simple or uniform specialization-injury relationship. Several studies reported greater injury occurrence or overuse-related injury among highly or early specialized athletes, whereas others found weaker or nonsignificant associations. Importantly, specialization often co-occurs with greater weekly training volume, longer annual participation, and sport-specific loading, making it difficult to determine whether specialization itself or its accompanying exposures explain observed differences in injury risk (Table 1).

**Table 1:** Injury and training-exposure evidence relevant to early sport specialization.

Study	Population/Sport	Specialization/Exposure	Injury Outcome/Denominator	Effect Estimate (95% CI)	Adjustment/Covariates	Interpretation
(17)	10,138 U.S. youth; mixed sports; prospective cohort	Single sport across fall, winter, and spring	Incident stress fracture, tendinitis, chondromalacia/patellofemoral syndrome, ACL tear, or osteochondral injury during prospective follow-up	Age-adjusted specialization: females HR 1.69 (1.41–2.03); males HR 1.50 (1.18–1.90). After vigorous-activity adjustment: females HR 1.31 (1.07–1.61); males HR 1.19 (0.90–1.58). Vigorous activity per additional hour/week: females HR 1.06 (1.05–1.08); males HR 1.04 (1.02–1.06).	Age; models additionally incorporated total weekly vigorous activity	Specialization estimates attenuated after volume adjustment, while activity volume remained independently predictive of injury.
(20)	2,011 athletes, ages 12–18; mixed sports	Low, moderate, or high specialization using 3-point scale; annual and weekly sport volume also assessed	Self-reported sport-related injury in previous year; participant-level injury history	High vs. low specialization: any injury OR 1.59 (1.26–2.02); overuse injury OR 1.45 (1.07–1.99). >8 months/year: upper-extremity overuse OR 1.68 (1.06–2.80); lower-extremity overuse OR 1.66 (1.22–2.30). Hours/week > age: any injury OR 1.34 (1.12–1.61).	Specialization analyses reported as independent of age, sex, and weekly organized sport volume	Supports both a specialization association and a separate workload association; specialization retained an independent association after weekly-volume adjustment.
(22)	716 athletes, ages 12–18; basketball, soccer, volleyball	High specialization; single-sport participation >8 months/year	Self-reported overuse injury in previous year; participant-level history	Volleyball: high specialization OR 2.3 (1.1–4.9); >8 months/year OR 2.0 (1.1–3.5). Basketball females vs. males: overuse injury OR 3.7 (2.1–6.6).	Sport-specific analyses; sex examined	Specialization was associated with overuse injury in volleyball but not basketball or soccer, demonstrating sport-specific heterogeneity.
(21)	551 male high-school baseball players; mean age 15.9 +/- 1.3 years; Alabama (200), California (188), and Michigan (163)	Low, moderate, or high specialization; >8 months/year baseball participation; pitcher status	Self-reported upper-extremity overuse injury during previous 12 months; throwing-arm health measured with Youth Throwing Score	High vs. low specialization: OR 3.77 (1.39–10.20), *p* = .009. Baseball >8 months/year: OR 2.03 (1.12–3.65), *p* = .019. Pitcher vs. non-pitcher: OR 2.11 (1.20–3.72), *p* = .010.	Multivariable regression; specialization–injury analyses accounted for geographic location/state, with specialization status, annual baseball participation (>8 months/year), and pitcher status evaluated as predictors of upper-extremity overuse injury.	High specialization remained associated with upper-extremity overuse injury after multivariable adjustment; annual exposure and pitcher status were also independent predictors.
(36)	159 male baseball players, ages 9–12; prospective follow-up 6.7 +/- 1.5 months	Research-based specialization classification	Prospectively documented shoulder/elbow injury; 21 injuries over 9,453 athlete-exposures; incidence 2.22/1,000 AEs	Specialized vs. nonspecialized: OR 6.7 (95% CI 0.4–118.0)	Unadjusted categorical analysis; position also examined	All prospectively observed arm injuries occurred among specialized athletes, but the confidence interval was extremely wide because of the small sample and zero injuries in the nonspecialized group.
(18)	178 elite youth football players in China; 88 boys, 90 girls	Early specialization vs. no ES; weekly volume, annual volume, training:leisure ratio, and maturity also measured	Self-reported musculoskeletal injury history	Any injury: ES OR 3.07 (1.16–8.09). Leg injury: weekly sport volume > age OR 2.05 (1.04–4.03). Foot injury: pre/early pubertal maturity OR 2.36 (1.02–5.47). Acute injury: ES OR 2.40 (0.99–5.84); weekly volume > age OR 2.55 (0.96–6.80).	Separate logistic models included specialization, weekly volume, annual volume, training:leisure ratio, and maturity	Specialization predicted any injury, whereas weekly volume was more clearly associated with leg injury. Several other estimates were imprecise or crossed the null.
(23)	238 Canadian junior-high students, ages 11–16	Low, moderate, high specialization based on year-round training, exclusion of other sports, and single-sport training	Self-reported musculoskeletal injury during previous 12 months; participant-level history	Moderate vs. low OR 1.94 (0.86–4.35); high vs. low OR 2.21 (0.43–11.37), both nonsignificant. Female sex OR 2.70 (1.69–4.35). Total annual sport hours OR 1.0010 per hour (1.0005–1.0020).	Multivariable model included specialization, sex, total annual sport hours, and clustering by school	Specialization was not independently associated with injury, while total sport exposure and female sex were.
(19)	1,171 injured youth athletes, ages 12.0–17.8, presenting to tertiary sports-medicine clinic	Exclusive high specialization; evolved high specialization; low/moderate specialization	Among already injured athletes: odds that presenting injury was overuse rather than acute	Exclusive high vs. low/moderate OR 1.56 (1.10–2.21); evolved high vs. low/moderate OR 1.36 (1.01–1.82). Female sex OR 1.75 (1.38–2.23). Among highly specialized athletes, individual vs. team sport OR 1.95 (1.17–3.24).	Three-group model adjusted for age and sex; highly specialized-group model additionally considered sport type, age intensive training began, and months of rest	Does not estimate population injury incidence. Among injured athletes, highly specialized athletes were more likely to present with overuse rather than acute injury.
(37)	143 elite/NCAA wrestlers; retrospective youth specialization history	Specialized before age 12 vs. age ≥12	Number of serious injuries sustained before college	Mean serious injuries: early specialization 1.14 vs. later specialization 0.60; *p* = .035	Group comparison; no adjusted OR reported	Retrospective evidence suggests greater serious-injury burden among wrestlers specializing before age 12, but no adjusted relative-risk estimate was reported.

AE, athlete-exposure; ACL, anterior cruciate ligament; CI, confidence interval; ES, early specialization; HR, hazard ratio; OR, odds ratio. Injury outcomes are not directly interchangeable across studies. Some studies report participant-level injury history, others prospective incident injury, and Murday et al. (19) evaluates injury type only among athletes already presenting with an injury. Adjusted estimates are prioritized where available. Specialization definitions also vary substantially across studies; effect estimates should be interpreted within each study's operational definition and exposure structure.

Field et al. (17) provides particularly informative evidence because of its large prospective cohort and explicit consideration of activity volume. Among 10,138 youth athletes, specialization was associated with injury in age-adjusted analyses among both females (HR = 1.69, 95% CI 1.41–2.03) and males (HR = 1.50, 95% CI 1.18–1.90). However, adjustment for weekly vigorous activity substantially attenuated these associations. The specialization estimate remained statistically significant among females (HR = 1.31, 95% CI 1.07–1.61) but not males (HR = 1.19, 95% CI 0.90–1.58). In contrast, each additional hour per week of vigorous activity remained independently associated with injury among females (HR = 1.06, 95% CI 1.05–1.08) and males (HR = 1.04, 95% CI 1.02–1.06) (17). These findings indicate that activity exposure may account for an important portion of the association attributed to specialization, although the residual association among females suggests that volume alone may not explain the relationship in all athletes.

Whatman et al. (23) similarly illustrates the importance of separating specialization from cumulative exposure. Neither moderate specialization (OR = 1.94, 95% CI 0.86–4.35) nor high specialization (OR = 2.21, 95% CI 0.43–11.37) was significantly associated with previous-year musculoskeletal injury after adjustment. In the same model, however, total annual sport hours remained positively associated with injury, as did female sex (OR = 2.70, 95% CI 1.69–4.35). The wide confidence interval surrounding the high-specialization estimate also illustrates the imprecision that characterizes some studies in this literature.

Other investigations indicate that specialization can retain an association with injury even when training exposure is considered. Post et al. (20) reported greater odds of any previous-year injury (OR = 1.59, 95% CI 1.26–2.02) and overuse injury (OR = 1.45, 95% CI 1.07–1.99) among highly specialized athletes compared with athletes with low specialization, independent of age, sex, and weekly organized sport volume. Training exposure nevertheless carried additional associations with injury. Participation in the primary sport for more than eight months per year was associated with upper- and lower-extremity overuse injury, and athletes whose weekly primary-sport hours exceeded their chronological age had greater odds of reporting an injury (20). Thus, specialization and workload should not necessarily be treated as competing explanations; in some populations, each may provide independent information about injury susceptibility.

#### 3.1.1 Sport-specific variation

Sport-specific findings further argue against a universal specialization effect. In a study of 716 adolescent basketball, soccer, and volleyball athletes, Post et al. (22) found that high specialization was associated with overuse injury among volleyball players (OR = 2.3, 95% CI 1.1–4.9), while comparable specialization associations were not observed in basketball or soccer. Volleyball participation for more than eight months per year was also associated with overuse injury (OR = 2.0, 95% CI 1.1–3.5). Among basketball athletes, females had substantially greater odds of overuse injury than males (OR = 3.7, 95% CI 2.1–6.6), further demonstrating that sex and sport may modify the apparent relationship between specialization and injury.

Baseball provides additional evidence that specialization occurs within a broader sport-specific exposure environment. Among 551 male high-school baseball players, high specialization was independently associated with previous-year upper-extremity overuse injury compared with low specialization (OR = 3.77, 95% CI 1.39–10.20) (21). However, playing baseball for more than eight months per year (OR = 2.03, 95% CI 1.12–3.65) and pitching (OR = 2.11, 95% CI 1.20–3.72) were also independently associated with overuse injury. These findings suggest that specialization status, annual exposure, and position-specific loading may each contribute to injury susceptibility.

Prospective evidence in younger baseball athletes points in a similar direction but remains imprecise. Arnold et al. (36) followed 159 male baseball players aged 9–12 years for approximately seven months and documented 21 shoulder or elbow injuries across 9,453 athlete-exposures. All observed arm injuries occurred among specialized athletes. The estimated association between specialization and injury was large (OR = 6.7), but the 95% CI was extremely wide (0.4–118.0), reflecting the limited number of events and absence of injuries in the nonspecialized group. The study therefore provides prospective evidence warranting further investigation but not a precise estimate of specialization-related risk.

Among elite youth football players in China, Li et al. (18) likewise found that early specialization was associated with greater odds of reporting any musculoskeletal injury (OR = 3.07, 95% CI 1.16–8.09). However, weekly sport volume exceeding chronological age was associated specifically with leg injury (OR = 2.05, 95% CI 1.04–4.03), while maturation status was associated with foot injury. Estimates for acute injury associated with specialization and weekly volume were elevated but imprecise and crossed the null. These findings again support a multifactorial interpretation involving specialization, workload, maturation, and sport-specific exposure.

#### 3.1.2 Injury type and injury denominators

Differences in injury definition and denominator are important when comparing studies. Murday et al. (19), for example, examined 1,171 athletes already presenting to a tertiary sports-medicine clinic. Compared with athletes with low or moderate specialization, those with exclusive high specialization had greater odds of presenting with an overuse rather than acute injury (OR = 1.56, 95% CI 1.10–2.21), as did athletes who evolved toward high specialization (OR = 1.36, 95% CI 1.01–1.82). These estimates should not be interpreted as demonstrating that highly specialized athletes were 56% or 36% more likely to become injured because the study population consisted entirely of injured athletes. Rather, specialization was associated with the type of injury among those seeking care.

Retrospective studies provide additional but methodologically distinct evidence. McDonald et al. (37) reported that elite and collegiate wrestlers who specialized before age 12 recalled a greater number of serious injuries before college than those specializing later (mean 1.14 vs. 0.60 injuries, *p* = .035). Such findings may provide insight into developmental histories but are susceptible to recall bias and should not be considered equivalent to prospective injury surveillance in current youth athletes.

Taken together, the injury literature suggests that specialization is associated with injury in several populations, but neither the magnitude nor independence of this relationship is consistent (Table 1). Training volume, annual participation, sport, sex, position-specific demands, maturation, previous injury, and injury ascertainment all influence interpretation. Current evidence therefore supports considering specialization as one component of a broader exposure profile rather than as a singular causal determinant of youth sport injury.

### 3.2 Training volume as a distinct but overlapping exposure

Training volume deserves separate consideration because it is conceptually related to, but not synonymous with, sport specialization. An athlete may specialize in one sport while maintaining a relatively moderate training schedule, whereas a multisport athlete may accumulate substantial total exposure across overlapping teams, seasons, and training environments. Similarly, year-round participation can occur without complete exclusion of other sports. These distinctions complicate studies that use specialization, annual participation, and workload interchangeably.

The available evidence suggests that training exposure can contribute to injury independently. Field et al. (17) found that weekly vigorous activity remained associated with injury after incorporating specialization into the models, while the specialization estimates attenuated. Whatman et al. (23) similarly identified total annual sport hours, but not specialization category, as an independent predictor of injury. Post et al. (20), in contrast, found evidence for both specialization and workload-related associations, while Post et al. (21) identified high specialization, annual baseball participation, and pitching as independent predictors of upper-extremity overuse injury in high school baseball.

Collectively, these studies suggest that training volume may explain part, but not necessarily all, of the relationship between specialization and injury. The extent of this overlap appears to differ according to sex, sport, outcome definition, and study population. Accordingly, recommendations focused exclusively on avoiding specialization may fail to identify highly exposed multisport athletes, while recommendations based solely on workload may overlook other characteristics of intensive single-sport environments.

The frequently cited recommendation that weekly organized sport hours should not exceed an athlete's chronological age should also be interpreted cautiously. Although several observational studies have used this threshold, the available evidence does not establish it as a universal biological boundary between safe and unsafe exposure. Field et al. (17), for example, demonstrated a graded relationship between vigorous activity and injury rather than a single threshold effect. Among females, even athletes participating in vigorous activity for 3–3.9 h per week less than their chronological age had greater injury risk than those participating at least four hours per week below their age (HR = 1.93, 95% CI 1.34–2.77). Thus, injury risk increased well below the commonly cited age-hours threshold. Workload recommendations are therefore better understood as pragmatic guidance that should be interpreted alongside sport demands, sex, maturation, competition exposure, previous injury, and recovery opportunity.

### 3.3 Movement, neuromuscular, and performance findings

Evidence concerning movement and neuromuscular outcomes is considerably more heterogeneous than the injury literature. Studies have evaluated different constructs, including balance, landing mechanics, lumbopelvic-hip movement, joint-coupling variability, coordination variability, and physical fitness, using different definitions of specialization and intensive participation. Consequently, the findings do not support a single generalized effect of specialization on movement competency or neuromuscular development (Table 2).

**Table 2:** Movement, neuromuscular, and performance findings relevant to sport specialization.

Study	Population/Sport	Exposure/Grouping	Task or Variable Measured	Observed Finding	Interpretation/Important Qualification
(38)	49 youth baseball and softball athletes (23 baseball, 26 softball); mean age 12.96 ± 2.32 years	Participation/training ≥8 months/year	Single-leg squat; lumbopelvic-hip movement/control	Group differences in trunk/lumbopelvic-hip performance were observed.	Exposure primarily represents annual training duration rather than the contemporary consensus definition of specialization. Findings should not be characterized simply as an ESS benefit; the authors discussed the observed characteristics as potentially training-related adaptations.
(39)	181 youth basketball players, ages 11.7–17	Earlier/single-sport specialization	Physical-fitness measures assessed across the season	Earlier specialization did not produce a consistent physical-fitness advantage.	Does not support a generalized physical-performance benefit from early specialization; interpretation is specific to the measured fitness outcomes and basketball population.
(23)	238 youth athletes, ages 11–16; mixed sports	Low, moderate, and high specialization	Fitness and functional performance measures	Minimal association was observed between specialization level and physical performance.	Provides little evidence for a broad physical-performance advantage associated with specialization.
(28)	355 athletes, ages 8–14; mixed sports	Sport-sampling history	Landing technique/neuromuscular landing task	Sport-sampling history was associated with differences in landing technique interpreted as more favorable neuromuscular control.	Represents a task-specific finding and should not be generalized to overall neuromuscular development or used as evidence that diversification universally prevents injury.
(29)	295 high-school athletes; mean age 15.6 ± 1.2 years; mixed sports	Single-sport vs. multisport participation	Y-Balance Test anterior reach asymmetry	Single-sport male athletes demonstrated greater anterior reach asymmetry than comparison groups.	Specific balance/reach finding; does not establish generalized neuromuscular impairment or a causal relationship with injury.
(27)	732 adolescent female basketball, soccer, and volleyball athletes; 366 sport-specialized and 366 multisport athletes	Specialization status	Hip-knee joint-coupling/coordination variability	Specialization-related differences in selected hip-knee coordination variability measures were observed.	Greater variability is not inherently beneficial or harmful. Its functional meaning depends on the task, coupling relationship, and association with performance or injury.
(26)	158 female adolescent athletes (79 specialized and 79 matched multisport), drawn from a database of 1,116; source database mean age 13.4 ± 1.8 years (not a study age range)	Specialization status considered across maturation	Lower-extremity biomechanics, including knee-abduction variables	Specialized athletes demonstrated larger post-pubertal changes in selected knee-abduction variables.	Demonstrates maturation-related biomechanical differences but does not independently establish injury causation.
(25)	91 adolescent female athletes, ages 13–16	Specialization level	Lower-extremity coordination variability	Highly specialized athletes demonstrated differences in coordination variability.	Coordination variability should not automatically be classified as a benefit or risk; clinical meaning is task dependent.
(40)	44 young female athletes	High vs. low sport specialization	Virtual-reality soccer-header task assessing center-of-gravity movement regularity; drop vertical jump assessment	Specialized athletes demonstrated lower center-of-gravity sample entropy during the virtual-reality header task than nonspecialized athletes (0.08 ± 0.02 vs. 0.10 ± 0.03), indicating more regular movement patterns.	Specialization was associated with greater movement regularity during the sport-specific virtual-reality task. The finding was task specific and does not establish that greater regularity represents impaired neuromuscular control or increased injury risk.
(24)	40 female youth soccer players; single-sport group mean age 15.05 ± 1.2 years; multisport group 15.32 ± 1.2 years	Specialization status	Landing Error Scoring System	Specialization did not significantly affect landing-error scores.	Provides a null biomechanical finding and reinforces heterogeneity across movement tasks and athlete populations.

This pattern is consistent with the systematic review by Zoellner et al. (41), which identified 13 studies examining sport specialization and movement competency. Four studies reported no significant specialization-related differences, seven identified differences in some but not all movement measures, and two retrospective studies found better movement competency among adults who had participated in multiple sports during high school. Multisport athletes showed more favorable jump mechanics or performance in several studies, but the overall literature did not show a consistent movement-competency phenotype by specialization status. Thus, the broader evidence supports interpreting specialization-related movement findings as task-specific rather than evidence of a uniform neuromuscular deficit. Studies examining coordination variability require particularly careful interpretation. DiCesare et al. (27) identified specialization-related differences in selected hip–knee joint-coupling variability measures among adolescent female athletes, while Bonnette et al. (25) reported differences in lower-extremity coordination variability according to specialization level. These findings should not be interpreted simply as showing either impaired or improved coordination. Movement variability can represent adaptive flexibility, alternative movement strategies, or reduced consistency depending on the task, coupling relationship, timescale, and outcome of interest. Barfield and Oliver (38) observed differences in single-leg-squat trunk control among youth baseball and softball players according to annual training exposure, while Riehm et al. (40) found lower center-of-gravity sample entropy during a virtual-reality soccer-header task among specialized female athletes. Both findings are task-specific and do not independently establish injury risk.

Maturation may further modify biomechanical associations. In a matched analysis of 158 female adolescent athletes (79 specialized and 79 multisport) drawn from a larger cohort, DiCesare et al. (26) reported specialization-related differences in selected lower-extremity biomechanical variables across maturation, including knee-abduction patterns. Such findings demonstrate that participation history may interact with developmental changes in movement mechanics, but they do not establish that the observed differences directly cause injury.

Evidence for a generalized performance benefit from early specialization is similarly limited. Soares et al. (39) did not identify a consistent physical-fitness advantage associated with earlier specialization among youth basketball players, while Whatman et al. (23) found minimal association between specialization level and physical-performance measures. These studies do not exclude the possibility that focused training improves highly sport-specific technical skills, but they provide little evidence that early specialization produces broad advantages in general physical fitness.

Overall, the movement, neuromuscular, and performance literature is better characterized as evidence of task- and sport-specific adaptation than as evidence of uniformly beneficial or detrimental movement development.

### 3.4 Psychological and psychosocial outcomes

The psychological evidence directly examining ESS is smaller and more heterogeneous than the injury literature. This distinction is important because broader concerns regarding burnout, anxiety, perfectionism, depression, disordered eating, and emotional distress are frequently discussed in relation to intensive youth sport but have not all been established as independent consequences of specialization itself. Accordingly, direct empirical ESS evidence should be distinguished from contextual youth-sport and review literature (Table 3).

**Table 3:** Psychological and psychosocial evidence relevant to early sport specialization.

Source	Source Type	Population/Exposure	Psychological or Psychosocial Outcome	Finding Supported by Source	Role in Current Review
(32)	Empirical longitudinal school-cohort study	One graduating school cohort followed annually from grades 7–12; annual survey *n* = 35–77; sport participation fell from 82% to 39%	Burnout	Specialization commonly emerged around the transition to high school. School burnout patterns changed across the middle-to-high-school transition but did not simply increase linearly with age. Burnout in sports did not increase with age	Longitudinal school-cohort evidence on participation and burnout trajectories; annual samples include students regardless of current sport participation. Does not establish ESS causation.
(42)	Empirical cross-sectional study	334 athletes, ages 12–18; sport participation characteristics	Athletic identity	Greater sport participation was associated with stronger athletic identity; team-sport participation was also associated with higher athletic-identity scores.	Direct empirical evidence relevant to athletic identity. Does not establish that ESS causes psychological distress, burnout, or impaired well-being.
(4)	Empirical parent-survey study	201 parent respondents (211 approached) describing their children's sport participation; children mean age 13.8 ± 3.0 years	Parental investment and specialization-related behavior	Parental investment in coaching and personal instruction was associated with specialization-related participation patterns.	Direct psychosocial/contextual ESS evidence. Useful for discussing environmental influences on specialization rather than as evidence of a mental-health outcome.
(43)	Empirical cross-sectional study	772 basketball players, ages 13–18; specialization and competitive participation volume	Time away from sport/recovery context	Specialized athletes reported limited time away from basketball.	Direct exposure/recovery-context evidence. Relevant to recovery opportunity but not direct proof of burnout, anxiety, or other psychological harm.
(30)	Review/professional synthesis	Pediatric sport-specialization literature	Burnout, stress, social and developmental concerns	Synthesizes psychosocial concerns associated with intensive youth sport participation and specialization.	Contextual synthesis only; should not be counted as an independent primary ESS outcome study.
(31)	Narrative/review literature	Youth and specialized athlete literature	Mental-health concerns	Discusses potential relationships among intensive sport environments, perfectionism, psychological distress, and related mental-health outcomes.	Contextual synthesis. Supports discussion of plausible concerns but should not be presented as direct evidence that ESS independently causes these outcomes.
(44)	Review/theoretical literature	Athletes broadly	Athlete burnout	Provides a conceptual framework for athlete burnout, including emotional/physical exhaustion, reduced accomplishment, and sport devaluation.	General burnout framework; not direct ESS evidence.
(45)	Empirical youth-sport study	Youth athletes; parental motivational climate/behavior	Motivation and enjoyment	Parental motivational climate and pressure were associated with athlete motivation and enjoyment.	Contextual youth-sport evidence. Relevant to the broader psychosocial environment but not direct evidence that ESS causes psychological outcomes.

Valenzuela-Moss et al. (32) provides longitudinal evidence from a school cohort surveyed annually from grades 7 through 12, with annual sample sizes of 35–77 students. Sport participation declined from 82% to 39% over follow-up, making it important to interpret burnout findings within a changing school cohort rather than a fixed athlete sample. Specialization commonly emerged around the transition to high school, while burnout trajectories varied across the middle-to-high-school transition rather than increasing uniformly with age. These findings support a developmental relationship among changing sport participation patterns and athlete burnout but do not establish a simple causal pathway in which specialization inevitably produces burnout.

Athletic identity represents another potentially relevant psychosocial construct. Christino et al. (42) found that greater sport participation was associated with stronger athletic identity among adolescent athletes, with team-sport participation also associated with higher athletic-identity scores. A strong athletic identity may provide belonging, motivation, and social connection, but an identity concentrated almost exclusively around sport could theoretically increase vulnerability when athletes experience injury, performance decline, deselection, or sport withdrawal. The latter interpretation remains contextual and should not be presented as a demonstrated adverse effect of ESS unless directly tested.

Family and environmental influences may also shape specialization. Padaki et al. (4) identified relationships between parental investment in coaching or personal instruction and specialization-related participation patterns. Such findings indicate that specialization develops within a broader social and economic environment rather than representing an athlete decision in isolation. However, parental investment should not automatically be interpreted as psychological pressure or harm without direct measurement of those outcomes.

Recovery opportunity may provide another link between intensive participation and well-being. Among adolescent basketball athletes, Meisel et al. (43) reported limited time away from basketball among specialized athletes. Reduced time away from sport may be relevant to both physical recovery and psychological detachment, but the observation does not itself demonstrate burnout or mental-health impairment.

Reviews and professional syntheses, including Brenner et al. (30) and Daley et al. (31), provide broader context regarding burnout, stress, perfectionism, and other psychosocial concerns associated with intensive youth sport environments. Similarly, general athlete-burnout frameworks help characterize emotional and physical exhaustion, reduced accomplishment, and sport devaluation. These sources are valuable for interpreting possible mechanisms but should not be counted as independent primary evidence that ESS causes these outcomes. Gustafsson et al. (44) provides a broader athlete-burnout framework, whereas Sánchez-Miguel et al. (45) examined parental behavior and youth sport enjoyment and amotivation; neither study independently establishes an ESS-specific effect.

Thus, psychological outcomes should be discussed more cautiously than injury outcomes. The available literature supports concern about the psychosocial environment surrounding intensive youth sport, but direct evidence isolating specialization from training load, competition pressure, parental influences, recovery, athletic identity, and other contextual factors remains limited.

### 3.5 Sport-specific loading as an evidence gap

Sport-specific mechanical demands may plausibly modify relationships among specialization, physical adaptation, and injury, but this hypothesis should be distinguished from direct ESS evidence. Sports differ considerably in repetitive loading patterns, movement requirements, dominant-limb use, impact exposure, and training structure. Consequently, specialization in baseball, gymnastics, tennis, golf, fencing, soccer, or basketball should not be assumed to represent a biomechanically equivalent exposure.

The baseball evidence demonstrates why sport-specific context matters. Pitching itself was independently associated with upper-extremity overuse injury in the high-school baseball study by Post et al. (21), while specialization and annual baseball participation were also associated with injury. This suggests that specialization may interact with the particular loading demands of an athlete's role rather than operating identically across sports.

Fencing represents a useful example of an understudied sport rather than established evidence of an ESS effect. Existing literature describes the physiological and movement demands of fencing and provides injury-surveillance information, but these studies generally do not compare athletes according to specialization status or age of specialization. Consequently, fencing-specific biomechanics or injury patterns cannot currently be interpreted as evidence that early specialization increases injury risk.

The same caution applies to broad categories such as “technical” or “asymmetrical” sports. Many sports contain substantial task-specific laterality, including sports not conventionally described as asymmetrical. Future studies should therefore quantify the relevant exposure directly rather than assigning sports to loosely defined biomechanical categories, including inter-limb force differences, dominance-dependent kinematics, bilateral asymmetry indices, repetitive loading, or task-specific movement volume—rather than assigning sports to loosely defined biomechanical categories.

Sport-specific loading is therefore best considered a potential effect modifier and research priority, not a demonstrated mechanism linking ESS universally to injury.

## 4 Discussion

This structured narrative review examined early sport specialization in relation to injury, training exposure, movement and neuromuscular adaptations, physical performance, and psychological well-being in youth athletes. Across these domains, the evidence supports a more nuanced interpretation of specialization than is often presented in youth-sport discussions. Higher or earlier specialization has been associated with injury in several populations, particularly for overuse-related outcomes, but these associations are neither universal nor readily attributable to specialization alone. Training volume, annual participation, sport, sex, maturation, position-specific demands, recovery, and other characteristics of the athlete's developmental environment frequently overlap with specialization and may independently contribute to observed outcomes.

The clearest implication of the current synthesis is therefore that specialization should not be conceptualized as a binary exposure that is inherently harmful or benign. Rather, its significance depends partly on the environment in which specialization occurs. An athlete training exclusively in one sport at a manageable volume with appropriate recovery may represent a substantially different exposure profile from an athlete participating in the same sport year-round at high weekly volume, across multiple teams, with limited recovery. Conversely, participation in multiple sports does not necessarily protect an athlete if overlapping seasons and teams produce excessive cumulative workload. This distinction has important implications for both interpretation of the existing literature and recommendations for youth athlete development.

### 4.1 Is specialization an independent risk factor or a marker of training exposure?

One of the central challenges in the ESS literature is the substantial overlap between specialization and training volume. Athletes who focus primarily on one sport often participate for more months of the year and accumulate greater sport-specific exposure. Some operational definitions of specialization incorporate year-round participation directly, further complicating attempts to separate specialization from workload statistically.

The injury literature illustrates this problem particularly well. Field et al. (17) found significant associations between specialization and injury among both females and males in age-adjusted models, but these estimates attenuated after weekly vigorous activity was incorporated. The association remained statistically significant among females but not males, while vigorous activity itself remained independently associated with injury in both sexes. Whatman et al. (23) similarly found that specialization was not independently associated with injury after adjustment, whereas total annual sport hours remained associated with injury.

These findings should not, however, be interpreted as demonstrating that training volume fully explains specialization-related injury. Post et al. (20) reported independent associations between high specialization and both overall and overuse injury while also identifying associations with workload-related exposures. Similarly, among high-school baseball players, high specialization, participation for more than eight months per year, and pitching were each independently associated with upper-extremity overuse injury (21).

Collectively, these studies suggest that specialization and workload may provide overlapping but nonidentical information. Greater exposure increases opportunities for repetitive tissue loading, while specialization may capture additional characteristics not represented by hours alone, including repetitive exposure to a narrower range of movement patterns, sport-specific competitive demands, reduced seasonal recovery, and the social structure surrounding intensive participation. The relative importance of these factors appears to vary among athlete populations.

Future studies should therefore avoid treating specialization and training volume interchangeably. Prospective models should measure sport-specific hours, total hours across all organized sports, months per year, competition exposure, number of simultaneous teams, recovery periods, previous injury, maturation, and specialization status independently. Such designs would allow investigators to determine whether specialization contributes risk beyond the exposure characteristics with which it commonly co-occurs.

### 4.2 Injury risk is sport-, sex-, and development-dependent

The substantial heterogeneity among sports is another reason to avoid universal conclusions regarding specialization. Post et al. (22), for example, identified an association between high specialization and overuse injury among volleyball players but not basketball or soccer athletes. In baseball, specialization occurs alongside highly specific throwing exposure, and pitching itself contributes independent information about upper-extremity injury risk (21). These findings suggest that the consequences associated with specialization cannot be separated entirely from the mechanical and competitive demands of the sport in which specialization occurs.

Sex may similarly modify associations. Field et al. (17) found that specialization remained associated with injury among females after adjustment for vigorous activity but not among males. Whatman et al. (23) identified female sex as an independent predictor of injury, while Post et al. (22) reported greater odds of overuse injury among female than male basketball athletes. These findings do not establish a single sex-specific mechanism but indicate that sex should not simply be included as a nuisance covariate and then ignored in interpretation.

Biological maturation may be equally important. Growth alters limb length, body mass, strength, coordination, tissue properties, and the mechanical demands imposed during sport participation. Li et al. (18) identified maturation-related differences in specific injury outcomes, while biomechanical studies suggest that specialization-related movement characteristics may differ across maturation. Chronological age alone may therefore provide an inadequate developmental measure.

Future research should be sufficiently powered to examine interactions among specialization, sex, maturation, sport, and workload rather than assuming that one specialization estimate applies equally to all youth athletes. These interacting influences are summarized in Figure 1, which presents a developmental-systems framework linking early sport specialization with training exposure, recovery, athlete characteristics, sport-specific loading, psychosocial context, and potential health and performance outcomes.

**Figure 1:**
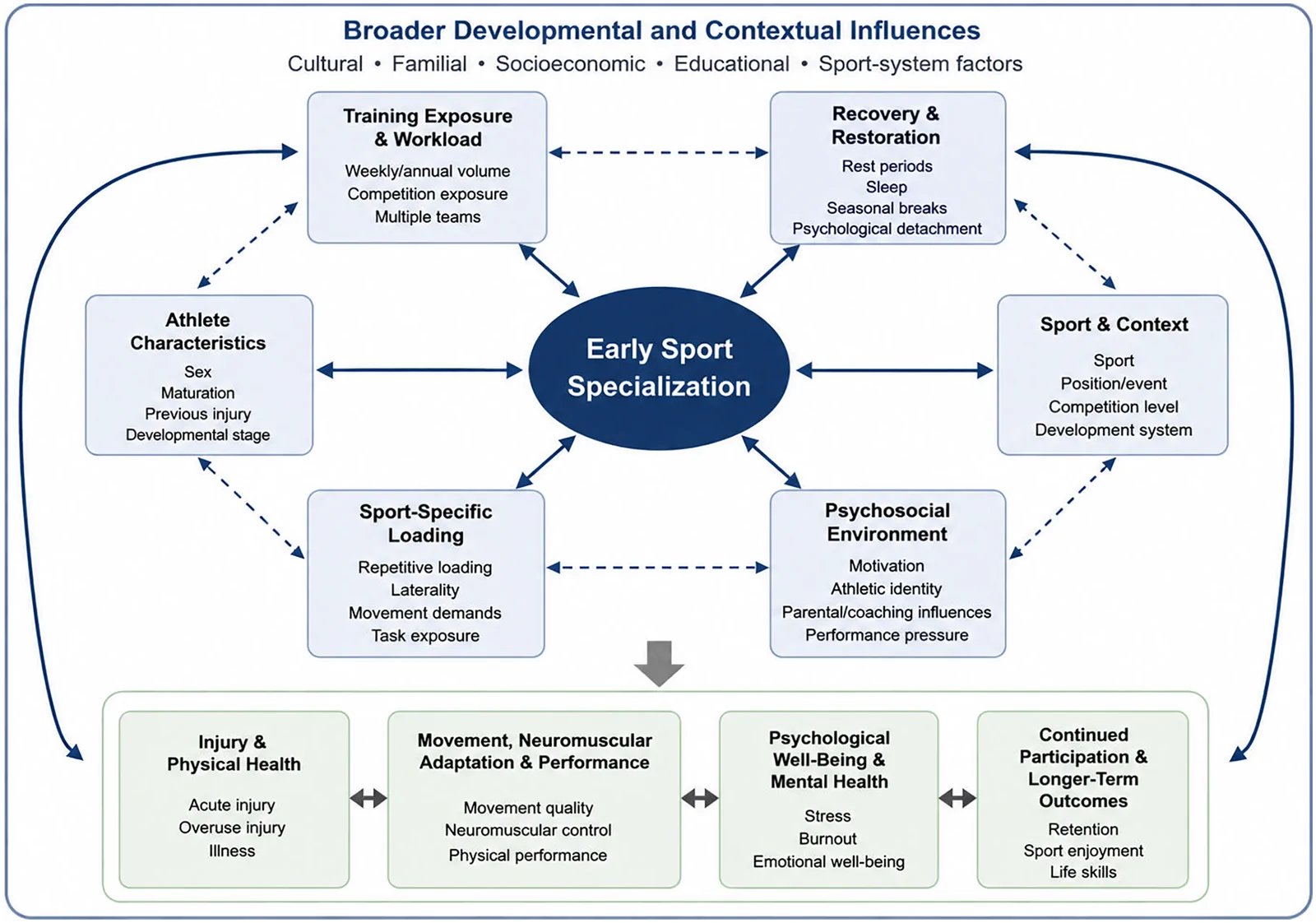
Developmental systems model of early sport specialization and youth athlete outcomes. Early sport specialization is conceptualized as occurring within a dynamic system of interacting athlete, training, sport-specific, recovery, psychosocial, and contextual factors rather than as an isolated exposure. Specialization interacts bidirectionally with training exposure and workload, sport-specific loading, recovery and restoration, athlete characteristics, psychosocial environment, and sport context, while broader developmental and contextual influences, including cultural, familial, socioeconomic, educational, and sport-system factors, shape these relationships over time. The combined balance of exposures and supports may contribute to multiple, interrelated outcomes involving injury and physical health, movement and neuromuscular adaptation and performance, psychological well-being and mental health, and continued sport participation and longer-term life outcomes. Pathways are not intended to imply deterministic causation; rather, the model illustrates the proposed interdependence of specialization and its correlated exposures and potential modifiers. Outcomes may also influence subsequent participation and developmental trajectories, emphasizing the dynamic and reciprocal nature of youth athlete development.

### 4.3 Reconsidering workload thresholds

The distinction between specialization and workload also has implications for commonly cited youth-sport recommendations. One frequently used guideline suggests that athletes should not participate in organized sport for more hours per week than their chronological age. The appeal of such a rule is understandable because it provides athletes, parents, coaches, and clinicians with a simple threshold.

However, the current evidence does not establish chronological age in hours per week as a universal biological boundary. Associations between workload and injury appear to occur across a continuum, and individual tolerance is likely influenced by sport, training intensity, competition exposure, maturation, previous injury, sleep, recovery, and other physical and psychosocial demands. Field et al. (17), for example, identified increasing injury risk with additional vigorous activity rather than demonstrating a single age-defined threshold; notably, elevated risk was observed among female athletes even when weekly activity remained three to four hours below chronological age. This finding directly challenges interpretation of the age-hours recommendation as a validated safety threshold.

The age-hours recommendation should therefore be regarded as a pragmatic screening heuristic rather than a validated cutoff separating safe from unsafe participation. Clinicians and coaches may reasonably use unusually high weekly volume as a signal to evaluate an athlete's overall training environment, but individualized workload and recovery assessment is preferable to reliance on a single numerical rule.

### 4.4 Movement and neuromuscular adaptations are not uniformly beneficial or harmful

The biomechanical literature further demonstrates the limitations of treating specialization as a unitary exposure. Studies have identified differences in balance, landing mechanics, joint coupling, coordination variability, and lumbopelvic-hip movement, but these findings do not converge on a single specialization-related movement phenotype. This interpretation aligns with Zoellner et al. (41), whose systematic review found no consistent overall difference in movement competency by specialization status. Although multisport participation was associated with more favorable jump mechanics or performance in several studies, findings varied substantially by movement task and outcome. Accordingly, neither specialization nor multisport participation should be assumed to produce a universally superior neuromuscular profile.

This is particularly important when interpreting movement variability. Greater variability is sometimes described as dysfunctional and lower variability as evidence of improved motor control; in other theoretical contexts, variability is viewed as an adaptive feature that permits flexible responses to changing task demands. Neither interpretation can be applied universally. The functional significance of variability depends on the movement being studied, the level at which variability is measured, the demands of the task, and whether the characteristic predicts subsequent injury or performance.

Accordingly, findings from DiCesare et al. (27) and Bonnette et al. (25) are more appropriately interpreted as specialization-related differences in specific coordination measures than as evidence of either improved or impaired neuromuscular function. Similarly, differences in Y-Balance Test performance, landing technique, or knee-abduction variables should not automatically be translated into injury risk without longitudinal evidence linking the measured characteristic to subsequent outcomes.

This distinction is particularly relevant during adolescence, when maturation itself produces substantial changes in movement mechanics. Longitudinal studies beginning before specialization and continuing across puberty would provide a stronger basis for determining whether observed biomechanical differences represent adaptive sport-specific development, potentially maladaptive loading patterns, or normal developmental variability.

### 4.5 Performance benefits of early specialization remain uncertain

An implicit justification for early specialization is that intensive sport-specific practice may increase the probability of later competitive success. However, existing systematic evidence does not establish early specialization as necessary for elite performance. Kliethermes et al. (16) reviewed the association between youth sport specialization and both career-focused and task-specific performance outcomes and concluded that specialization was not required to achieve elite athletic success. Task-related outcomes, including neuromuscular control, anterior reach asymmetry, and physical-performance measures, also did not consistently differ according to specialization status. The authors emphasized, however, that the available literature was limited in both volume and methodological rigor. Accordingly, the evidence is better interpreted as failing to demonstrate a generalized performance advantage from early specialization than as proving that later specialization or multisport participation is universally superior.

This does not mean that sport-specific practice lacks value. Expertise clearly requires sustained practice, and certain sports may reward earlier acquisition of highly specific technical skills. However, the concept of deliberate practice should not be conflated with a requirement for early exclusive participation. Ericsson et al. (11) emphasized sustained purposeful practice in expertise development but did not establish a universal “10,000 h rule” or demonstrate that young athletes must abandon other sports to achieve elite performance.

This interpretation also complements evidence comparing sport sampling and specialization. Carder et al. (15) found a lower injury risk among youth athletes who sampled multiple sports than among athletes who specialized. Although that finding addresses injury rather than subsequent athletic performance, it is relevant to developmental decision-making because the potential health trade-offs of early specialization become harder to justify when an accompanying general performance advantage has not been established. Sampling should nevertheless not be interpreted as inherently protective or universally optimal, particularly when participation across multiple sports produces high cumulative workload.

Athlete-development models likewise permit more than one pathway. The Developmental Model of Sport Participation recognizes sampling and recreational pathways as well as earlier specialization in selected contexts, while Long-Term Athlete Development frameworks provide stage-based guidance rather than proof of a single optimal developmental sequence. Neither framework should be interpreted as demonstrating that diversification is universally superior or that early specialization is universally necessary.

The more useful question may therefore be when increasing sport-specific commitment becomes developmentally appropriate for a particular athlete and sport, rather than whether specialization should always be delayed to a predetermined age.

### 4.6 Psychological well-being and the intensive sport environment

The psychological evidence requires even greater interpretive caution than the injury literature. Burnout, athletic identity, performance pressure, parental involvement, reduced time away from sport, and related psychosocial characteristics are relevant to intensive youth-sport environments, but relatively few studies isolate specialization as an independent exposure.

Valenzuela-Moss et al. (32) provides useful longitudinal evidence concerning specialization and burnout trajectories across adolescence, while Christino et al. (42) demonstrates relationships between sport participation and athletic identity. Other studies identify parental and recovery-related characteristics that may shape the environment surrounding specialization. These findings support consideration of psychological well-being in athlete-development decisions but do not demonstrate that specialization inevitably produces burnout, anxiety, depression, perfectionism, or other adverse mental-health outcomes.

Athletic identity illustrates the need for balanced interpretation. Strong identification with sport may support belonging, motivation, social connection, and persistence. At the same time, a highly exclusive athletic identity could plausibly create vulnerability when participation is interrupted by injury, deselection, or retirement. Whether specialization increases this vulnerability, and under what circumstances, remains insufficiently established.

Similarly, reduced time away from sport may have both physical and psychological implications. Recovery involves more than tissue repair; time away from structured sport may provide opportunities for social development, psychological detachment, alternative activities, and autonomous recreation. Prospective studies should therefore evaluate workload and recovery alongside burnout, motivation, identity, enjoyment, sleep, and mental-health outcomes.

### 4.7 Sport-specific loading and understudied sports

Sport-specific loading represents an important but incompletely studied dimension of the specialization literature. Broad categories such as “technical” or “asymmetrical” sports do not currently constitute coherent empirical exposure groups because sports within these categories differ substantially in movement patterns, loading characteristics, competitive structures, and developmental pathways. Moreover, many sports not conventionally classified as asymmetrical contain substantial task-specific limb dominance.

Fencing provides an instructive example. Existing studies describe its physiological demands, repetitive stance and lunge patterns, and injury epidemiology, but direct studies evaluating youth fencing specialization and subsequent injury or developmental outcomes remain lacking. The available fencing literature therefore identifies a research opportunity, not evidence that ESS has already been demonstrated to increase injury risk in fencers.

Future research should replace categorical labels such as “asymmetrical sport” with direct measures of the proposed exposure. Depending on the research question, these might include inter-limb force differences, dominance-dependent kinematics, unilateral loading volume, bilateral asymmetry indices, or cumulative sport-specific movement exposure. Such approaches would allow investigators to test whether specific loading characteristics interact with specialization rather than assuming that sport category itself determines risk.

### 4.8 International and developmental context

The meaning of specialization also depends on the structure of the youth-sport system. In the United States, pressures toward earlier specialization may arise through travel teams, private instruction, collegiate recruitment, scholarship aspirations, and increasingly competitive school and club environments. These pathways should not be assumed to characterize youth sport internationally.

In other countries, elite development may occur predominantly through club academies, sport federations, national development systems, or other structures in which focused participation begins at different developmental stages. Consequently, recommendations regarding “delaying specialization” may have different practical implications across countries and sports.

The international diversity of the literature reviewed here is therefore both a strength and a source of heterogeneity. Rather than assuming that a universal age of specialization can be prescribed, athlete-development recommendations should consider the sport, developmental pathway, training environment, and cultural context in which specialization occurs.

### 4.9 Practical implications

The current evidence supports moving beyond a binary recommendation to either “specialize” or “do not specialize.” Instead, decisions concerning increasing sport-specific commitment should consider the athlete's complete developmental and training environment.

These practical considerations should also be interpreted in relation to existing organizational guidance, which itself reflects both areas of consensus and substantial uncertainty. Herman et al. (46) systematically reviewed health-organization recommendations concerning youth sport specialization and identified considerable variation in their content, specificity, and evidentiary basis. Recurring recommendations included monitoring athlete well-being, providing access to qualified coaching, encouraging multisport participation, limiting excessive early organized training, and improving parental awareness. Tenforde et al. (47) extended this question by evaluating 23 youth-sport guidelines from sport organizations and governing bodies across soccer, basketball, ice hockey, swimming, and multisport organizations. Consensus was incomplete. Most organizations emphasized developing fundamental skills and access to qualified coaching, and multisport participation was commonly encouraged, but recommendations on training volume, rest, specialization timing, and other aspects of athlete development varied within and across sports. Notably, none of the evaluated organizational guidelines provided a specific strategy for monitoring athlete well-being. Taken together, these reviews demonstrate that current guidance converges on broad principles of developmentally appropriate participation but does not establish a single evidence-based prescription for when athletes should specialize or how much training is universally appropriate. The present synthesis is consistent with this individualized approach: specialization decisions should consider cumulative workload, recovery opportunity, maturation, sport-specific demands, athlete goals, and physical and psychological well-being, rather than being governed by specialization status or a single numerical threshold. Table 4 summarizes the practical implications of the reviewed evidence, emphasizing individualized assessment of specialization, training exposure, recovery, developmental readiness, and athlete well-being.

**Table 4:** Integrated summary of findings, qualifications, and research priorities.

Domain	Overall Pattern in the Reviewed Literature	Key Qualifications	Major Research Priority
Sport specialization and injury	Several studies report associations between higher or earlier specialization and injury, particularly overuse-related outcomes.	Associations are not universal and vary by sport, sex, specialization definition, injury definition, denominator, and study design. Most evidence is observational.	Prospective studies using standardized specialization definitions, standardized injury surveillance, and clearly defined exposure denominators.
Training volume and injury	Higher weekly or annual sport exposure is repeatedly associated with injury, and some studies identify workload-related associations independent of specialization.	Training volume is correlated with specialization and may explain or modify part of the observed specialization-injury relationship. In Field et al. (17), specialization estimates attenuated after adjustment for vigorous activity volume.	Models simultaneously measuring specialization, total and sport-specific workload, annual exposure, competition exposure, and recovery.
Specialization independent of workload	Evidence for an independent specialization effect is mixed. Some adjusted studies retain an association, whereas others show attenuation or nonsignificant estimates after accounting for activity exposure.	Post et al. (20) and Post et al. (21) retain specialization-related associations in adjusted analyses, whereas Field et al. (17) and Whatman et al. (23) illustrate attenuation or lack of an independent association in some groups.	Longitudinal studies specifically designed to separate specialization from training volume, year-round participation, competition level, and recovery.
Sex, sport, and developmental differences	Associations vary across sex, sport, and developmental context rather than supporting a single universal specialization effect.	Field et al. (17) reported different volume-adjusted estimates for females and males and sport-specific heterogeneity. Maturation is inconsistently measured across the broader literature.	Adequately powered sex-, sport-, age-, and maturation-stratified prospective studies.
Movement and neuromuscular adaptations	Specialized or intensively trained athletes demonstrate task-specific differences in balance, coordination variability, joint coupling, landing mechanics, and other movement variables.	No consistent direction of effect is evident. Greater or lower movement variability cannot automatically be classified as adaptive, maladaptive, protective, or injurious.	Longitudinal studies linking specific biomechanical adaptations to subsequent injury and performance outcomes.
Physical performance	Current evidence provides limited support for a generalized physical-performance advantage from early specialization.	General fitness outcomes are not equivalent to sport-specific skill acquisition. Different sports may have different developmental pathways and performance demands.	Long-term studies linking childhood participation and specialization histories with objective later performance outcomes.
Psychological and psychosocial outcomes	Burnout, athletic identity, recovery opportunity, parental influences, and related psychosocial factors are relevant to intensive youth-sport environments.	Direct ESS-specific psychological evidence is comparatively sparse and heterogeneous. Reviews and broader youth-sport studies should not be treated as independent evidence that ESS causes mental-health outcomes.	Prospective studies jointly measuring specialization, workload, recovery, burnout, mental health, motivation, identity, and social context.
Sport-specific repetitive or lateralized loading	Sport-specific loading may plausibly modify relationships among specialization, physical adaptation, and injury.	Biomechanical or injury-surveillance studies in fencing and other sports do not demonstrate an ESS effect unless specialization itself is measured. Loosely defined categories such as “technical” or ‘asymmetrical’ sports should not substitute for quantified loading or laterality.	Direct specialization studies in understudied sports incorporating quantitative loading, dominance/laterality, and inter-limb measures.
Workload recommendations	Monitoring cumulative workload and ensuring adequate recovery are broadly supported as practical considerations in youth sport.	The commonly cited recommendation that organized sport hours per week should not exceed chronological age is a pragmatic heuristic rather than a validated universal biological threshold.	Dose-response studies establishing sport-, sex-, maturation-, and recovery-specific workload relationships and clinically useful thresholds.
Overall interpretation	Specialization status may identify a higher-exposure developmental environment associated with selected adverse outcomes, but it is not a sufficient standalone explanation.	Workload, recovery, maturation, previous injury, competition level, sport-specific demands, sex, and psychosocial environment may confound or modify observed associations.	Multivariable longitudinal research capable of estimating independent and interactive effects and identifying which athletes are most vulnerable or resilient.

For clinicians, coaches, parents, and sport organizations, several practical considerations emerge from the literature:

- Monitor total training and competition exposure, including participation across multiple teams and sports, rather than counting only hours in the primary sport.
- Preserve meaningful recovery periods within weeks and across the annual training cycle.
- Consider previous injury, sex, maturation, position or event, and sport-specific loading when evaluating workload tolerance.
- Avoid assuming that multisport participation is inherently protective if cumulative exposure remains high.
- Avoid assuming that specialization is inherently harmful when training volume, recovery, and athlete well-being are appropriately managed.
- Monitor enjoyment, motivation, burnout symptoms, sleep, and psychological recovery alongside physical health.
- Reassess increasing specialization as athletes mature rather than treating an early participation decision as irreversible.
- Consider the athlete's goals and preferences rather than letting scholarship, recruitment, parental, or organizational pressures automatically determine the pathway.

Taken together with existing organizational guidance, these considerations shift emphasis from rigid prohibition or universal thresholds toward developmentally appropriate exposure management.

### 4.10 Strengths and limitations

A strength of this structured narrative review is its integration of injury, workload, movement and neuromuscular, performance, and psychological evidence rather than treating these outcomes independently. Particular attention was given to distinguishing specialization from training exposure and to identifying circumstances in which adjusted estimates differ from unadjusted associations. The review also distinguishes direct ESS evidence from contextual sport-specific, biomechanical, and psychological literature, reducing the risk of attributing outcomes to specialization when specialization itself was not measured.

Several limitations should be acknowledged. First, this is a structured narrative review rather than a systematic review. The structured search was conducted primarily in PubMed/MEDLINE, and literature indexed exclusively in other databases may therefore have been missed. The review was not prospectively registered, and it did not apply a formal PRISMA-based selection process, systematic risk-of-bias assessment, or certainty-of-evidence framework. Conclusions should consequently be interpreted as an integrative synthesis rather than an exhaustive systematic estimate of the literature.

Second, the underlying evidence is heterogeneous. Definitions of specialization differ substantially among studies and sometimes incorporate year-round participation or training duration, producing conceptual overlap between specialization and workload. Injury definitions and denominators also vary, ranging from retrospective participant-level injury history to prospectively documented injuries per athlete-exposure and injury type among already injured clinical populations. Direct numerical comparisons across such studies are therefore inappropriate.

Third, much of the evidence is cross-sectional or retrospective, limiting causal inference. Recall bias may affect both specialization histories and previous injury reporting, while differential access to athletic trainers or sports-medicine services could influence injury ascertainment. Specialized athletes participating in highly organized sport environments may have injuries documented more readily than recreational athletes, although competitive athletes may also underreport symptoms to avoid participation restrictions.

Fourth, potentially important confounders and effect modifiers are inconsistently measured. These include previous injury, biological maturation, sleep, recovery, competition level, socioeconomic resources, coaching environment, and total participation across multiple sports. Psychological evidence directly examining specialization is particularly limited.

Finally, the international diversity of youth sport systems limits the applicability of universal recommendations. Developmental pathways that are feasible within U.S. school and collegiate structures may not translate directly to academy- or federation-based systems elsewhere.

These limitations reinforce the need for prospective research using standardized definitions and multidimensional exposure measurement.

### 4.11 Future research directions

The next generation of ESS research should move beyond simple comparisons of “specialized” and “nonspecialized” athletes. Longitudinal studies should begin before specialization occurs whenever possible and follow athletes across maturation. Repeated measures should capture specialization status, weekly and annual training volume, competition exposure, number of teams, recovery, sleep, previous injury, maturation, psychosocial characteristics, and sport-specific loading.

Three questions are particularly important. First, does specialization independently predict injury or psychological outcomes after cumulative exposure and recovery are adequately measured? Second, which athlete and sport characteristics modify that relationship? Third, are there combinations of specialization and workload that permit sport-specific development without increasing adverse outcomes?.

Biomechanical studies should similarly move from cross-sectional group differences toward prospective prediction. Rather than assuming that greater asymmetry, greater variability, or a particular movement pattern represents risk, investigators should determine whether those characteristics predict future injury or performance.

Psychological research should distinguish specialization from the broader intensive-sport environment and incorporate validated measures of burnout, anxiety and depressive symptoms, athletic identity, motivation, enjoyment, autonomy, sleep, and social support.

Finally, understudied sports, including fencing and other sports with distinctive repetitive or lateralized loading, represent useful opportunities for hypothesis-driven prospective research. Such studies should measure both specialization and the proposed biomechanical exposure directly.

## 5 Conclusions

Across the literature reviewed, higher or earlier sport specialization was associated with greater injury risk in several youth athlete populations, particularly for overuse-related outcomes, although the relationship was not consistent across sports or athlete groups. Associations varied according to sport, sex, developmental stage, outcome definition, and study design and may be influenced by training volume, annual participation, recovery, previous injury, competition level, and sport-specific demands.

Training exposure appears particularly important. In some studies, adjustment for activity volume markedly attenuates specialization-related injury estimates, whereas other investigations identify independent associations for both specialization and workload. Thus, current evidence supports neither the conclusion that specialization alone drives injury nor the conclusion that workload completely explains specialization-related risk.

Evidence for generalized biomechanical or performance consequences is less consistent, and direct psychological evidence remains comparatively limited. Movement differences associated with intensive participation should not automatically be classified as adaptive or maladaptive, and broader mental-health concerns arising within intensive youth sport should not be attributed specifically to specialization unless specialization itself has been evaluated.

The practical implication is therefore not a universal prohibition on specialization. Instead, athlete-development decisions should consider when, how, and under what conditions increasing sport-specific commitment occurs. Monitoring cumulative workload, providing adequate recovery, accounting for maturation and previous injury, preserving psychological well-being, and considering sport-specific demands may be more informative than specialization status alone.

Future prospective research capable of disentangling specialization from its correlated exposures is needed to identify which combinations of sport-specific commitment, workload, recovery, and developmental context optimize both athlete health and long-term participation.

## Data Availability

The original contributions presented in the study are included in the article/Supplementary Material, further inquiries can be directed to the corresponding author/s.
